# Schoolbook Texts: Behavioral Achievement Priming in Math and Language

**DOI:** 10.1371/journal.pone.0150497

**Published:** 2016-03-03

**Authors:** Stefan Engeser, Nicola Baumann, Ingrid Baum

**Affiliations:** Department of Psychology, University of Trier, Trier, Germany; University of Groningen, NETHERLANDS

## Abstract

Prior research found reliable and considerably strong effects of semantic achievement primes on subsequent performance. In order to simulate a more natural priming condition to better understand the practical relevance of semantic achievement priming effects, running texts of schoolbook excerpts with and without achievement primes were used as priming stimuli. Additionally, we manipulated the achievement context; some subjects received no feedback about their achievement and others received feedback according to a social or individual reference norm. As expected, we found a reliable (albeit small) positive behavioral priming effect of semantic achievement primes on achievement in math (Experiment 1) and language tasks (Experiment 2). Feedback moderated the behavioral priming effect less consistently than we expected. The implication that achievement primes in schoolbooks can foster performance is discussed along with general theoretical implications.

## Introduction

### Behavioral priming effects

Semantic priming studies demonstrate the influence of textual stimuli on behavior for various domains. Reading words related to health behavior reduced the number of snacks taken [1], reading words such as “aggressive” and “rude" led subjects to interrupt the experimenter more quickly and frequently [2], and semantic primes of social groups associated with helping behavior (e.g., nurses) led to more helping behavior [3]. Of special interest for our work, reading words related to the achievement domain like “win,” “master,” or “achieve” led to higher performance. This has been shown by Bargh, Gollwitzer, Lee-Chai, Barndollar, and Trötschel [4] and replicated by others (e.g, [5–8]). Furthermore, the considerable practical importance of achievement priming effects has been shown for pictorial achievement primes in natural settings at the workplace (cf. [9]).

Taking the results of these studies, such prime-to-behavior effects of semantic primes should influence behavior in daily life. In educational settings, students are constantly exposed to semantic stimuli from textbooks, teacher’s instruction, and peers. Accordingly, one could regard books (and others) as a source of daily semantic stimuli. For books this would imply that books containing more achievement primes foster performance, and this should cumulate to higher academic performance when this occurs over a longer time period. Evidence for such an effect was provided by Engeser, Rheinberg, and Möller [10]: They observed that two German federal states with marked differences in scores on nation-wide examinations of academic performance also had reliable differences in achievement imagery of the schoolbooks used. Additionally, a recent correlational study showed higher achievement imagery in 4^th^ grade school books was positively related to higher academic performance in 4^th^ grade [11].

In explaining the semantic behavioral priming effects it is assumed that the semantic stimuli (i.e., primes) activate mental representations. Beyond this basic assumption, the specific mechanisms of how primes affect behavior is the object of controversial theoretical positions. The perception-behavior link hypotheses [2, 12, 13] propose a direct link of semantic information to behavior. The behavior associated with the semantic information will be directly executed (like seeing a smiling person will make us smile). A more flexible link of semantic information to behavior is proposed by Bargh et al. [4, 14]. They maintain that semantic primes activate a goal state and that the goal will subsequently guide behavior in the same manner as intentionally set goals. In the achievement domain, the activated goal would be the “goal of doing well” [4]. That a primed goal or a mental content may not always lead to specific behavior responses seems obvious and is of growing theoretical interest to understand prime-to-behavior effects. On an empirical level, various moderators of the prime-to-behavior effect have been identified, providing evidence that primed mental contents do not always lead to the respective behavior.

The importance of individual differences is portrayed by Wheeler, DeMarre, and Petty [15, 16] in their active self-account. They propose that primes alter information of the active self-concept and that the active self-concept guides behavior. Smeesters, Wehheler, and Kay [17] additionally proposed that primes alter the perception of others or the situation which moderates the response to this person or situation. That primes can influence behavior by changing the interpretation of situations was also proposed by the situated inference model [18], an aspect discussed below in respect to the role of context on prime-to-behavior effects. Further, primes activate “matching behavior” such as reading the word “celebrity” stimulating reaction speed to words like “adore” and “clap” (instead of acting like a star as a direct prime-to-behavior account may assume) [19]. Comparably, the same primes lead to different behavioral responses depending on what action was allowed by the situation (i.e., situational contingencies) [20]. For the achievement domain, Hart and Albarracin [7] found the context to alter the effect of achievement primes, and Engeser and Baumann [21] found evidence that achievement priming effects are mediated by outcome-expectancies.

A more complete understanding of behavior priming effects seems of central importance because despite many positive findings in this field of research, priming effects were recently called into question [22]. In a direct replication, Harries, Coburn, Rohrer, and Pashler [23] failed to replicate the achievement priming effect of Bargh et al. [4] mentioned above. In a direct and conceptual replication, Doyen, Klein, Pichon, and Cleeremans [24] failed to replicate the finding that reading words related to old age slowed down walking speed [2, 25]. They found that the walking speed effect occurred only when the experimenter expected the primed subject to walk slower. Shanks et al. [26] also failed to replicate a classical study on priming intelligent behavior. They argued that traditional priming research on lexical or semantic processing had assumed a narrow context-specific priming effect that does not generalize very broadly. This would imply that priming effects are sensitive to (subtle) experimental conditions or context factors in general. Stated differently, primes affect the active mental content, but whether they affect specific behaviors depends on the given context.

### The role of context

Gawronski and Cesario [27] outlined the importance of context effects of behavioral priming and highlighted the fact that this aspect has been widely ignored by previous behavior priming research. Contexts specify which actions are prepared and executed; thus, ignoring context would assume that priming is merely highly automatic and inflexible. In line with Gawronski and Cesario [27], Jonas and Sassenberg [28] showed that context information does alter the activation and execution of response behavior. The activation of behavioral response in respect to the context (e.g., a flexible response) was also shown by Cesario, Plaks, Hagiwara, Navarrete, and Higgins [20]. They found opposite behavioral readiness when the priming experiment was run in the lab or in an open field. In the outline of their situation interference model, Loersch and Payne [18] also argued and provided empirical evidence that primes often do not cause direct effects but instead alter the perception and interpretation of situational features.

In the achievement domain, Stajkovic, Locke, and Blair [29] showed that explicit goal instructions moderated the priming effect. Only in the conditions of difficult and do-best goals (i.e., achievement context), but not in the condition with easy goals (i.e., no achievement context), did priming lead to better performance on the same task. Crusius and Mussweiler [5] found that comparative mindsets moderated achievement priming effects: Whereas a similarity focus led to assimilation to an achievement prime, a difference focus resulted in contrast. Hart and Albarracin [7] revealed that semantic behavioral priming effects varied as a function of task instructions and individual differences in achievement motivation. When subjects were informed that the tasks were diagnostic of ability (i.e., achievement context), priming effects (i.e., a more frequent choice for an achievement task and higher performance) were found among individuals chronically high in achievement motivation. When tasks were introduced as a fun game (i.e., no achievement context), priming effects were found among individuals chronically low in achievement motivation. Taken together, the reviewed findings suggest that achievement priming leads to higher performance if the context matches the semantic prime content.

### The present research

We used excerpts from schoolbooks to determine if primes foster achievement when embedded in running text and, thus, assess the potential practical relevance of semantic achievement primes. Specifically, we expected an achievement priming effect for achievement primes in running text. We did not expect a strong priming effect. The behavioral priming effect may be less strong than the first achievement priming studies suggested [6], and the recent result on the reproducibility of psychological science indicate that effects are less strong in replication studies [30]. Additionally, we took into account that embedded achievement primes in running text might possibly weaken the priming effects. Further, and as outlined above, priming effects were assumed to be moderated, leading to at least a reduced main effect of priming. In order to detect even weak priming effects, we increased power by using a lager sample size. This also gave us the power to detect moderation effects.

Beside the main effect of achievement priming, we looked for moderators of the priming effect in order to more fully understand the prime-to-behavior effect and its practical implications by varying the achievement context. This was accomplished by providing subjects no feedback or feedback. Feedback is a central aspect of achievement motivation and behavior as it provides information about success or failure and, in interplay with other factors, influences performance [31, 32]. Thus, feedback is context information which makes the task more achievement related, and we expected semantic priming effects to be stronger in the feedback condition. In the feedback condition, we differentiated between social and individual reference norms (cf. [33, 34]). In respect to priming research, there are some indications that priming effects are stronger under social reference norms, as the presentation of achievement tasks as an ability test implies a social reference norm by comparing one’s own performance with those of others [7, 35]. However, behavioral achievement priming effects have also been observed under do-best goals [29]. Thus, we expected an individual reference as well as a social reference norm to provide a context in which priming effects unfold. Taken together, we expected (1) a semantic priming effect of achievement primes in running text, (2) a stronger priming effect in the feedback conditions, and (3) that the stronger effect in the feedback conditions holds regardless of the type of feedback (i.e., social or individual reference norm).

We selected text excerpts from 9^th^ grade math (Experiment 1) and language arts (Experiment 2) books. Performance was measured with arithmetic tasks in the first and with anagram tasks in the second experiment. Beyond some general associations of schoolbook excerpts and task, the texts were not related to the experimental task. We selected different texts for neutral and achievement texts for our experimental manipulation. This is somewhat problematic, as we did not strictly vary semantic achievement content. However, we decided to do so in order to simulate a natural priming manipulation because one of our intentions was to examine practical implications of semantic priming. If we would have selected passages of text with high achievement content and deleted this content, factors inherent in excerpts, such as the context or some other hints, may have still implied some achievement content. To avoid this, a strong modification of the textual passages would have been necessary. This was not feasible for practical reasons, as some achievement texts like being world champion in SMS fast writing (see section Priming materials 1 below) could have hardly been transformed from an achievement text to a completely neutral text.

## Experiment 1

### Method 1

#### Subjects and Design 1

One hundred sixty-nine subjects took part in the study, which was conducted at a university in Germany. For two subjects, we encountered software problems, four subjects had problems understanding the calculations (subjects were not able to apply the rules after the practice trials as described below), two subjects were aware of the experimental manipulation and two subjects did not work seriously on task (i.e., typing random numbers; one in the neutral and one in the achievement condition). These subjects were excluded from the analysis. Of the remaining 159 subjects (aged 19–34, *M* = 23.34, *SD* = 2.76), 109 were women. All subjects were students (five majoring in psychology) and were paid for participation (15€).

We have complied with APA ethical standards in the treatment of our human sample. We informed participants that the data are used for scientific purposes only, the investigation is conducted fully anonymously, and with their participation they consent to the use of the data for scientific purposes. We explicitly emphasized that subjects could stop and object the use of the data at any time without any personal consequences. We used minor experimental manipulation with everyday stimuli and assessed only basic biographical information (age, gender, level of education). Priming effects are expected to be short-lived, and even if the effect prolonged the experimental setting, there would be no reason to expect any harm to the subjects. Based on these aspects, we saw no need for formal ethical approval by an ethics committee beyond the approval of the experiments by the German Research Foundation.

We employed a 2 (neutral vs. achievement prime text content) x 3 (no feedback vs. social reference norm vs. individual reference norm) experimental between-subjects design. Performance was repeatedly measured in 16 trials.

#### Procedure 1

The experiment was conducted in a separate, small room. Instructions on the computer informed subjects that the study was about language processing and concentration. After completing an implicit achievement motive measure, the Operant Motive Test [36], subjects were introduced to the arithmetic task and completed a practice block of six tasks (data for the implicit achievement motive are not presented here; analyses revealed that the implicit achievement motive did not significantly moderate priming effects). If they solved less than four of the six tasks correctly, they were again provided with the instructions for the arithmetic tasks and had to solve the practice block again. If they failed again, they had to inform the experimenter who explained the task verbally, and then they started the practice blocks again. Next, subjects completed two blocks of the experimental arithmetic task to assess baseline performance (subjects had 90 seconds to solve as many tasks as they could). After the baseline performance, they were informed that a text would be presented at some point within the 16 experimental blocks of arithmetic tasks. They were also informed that they should carefully read the text and that the text would be relevant later in the experiment. Subjects in the neutral priming condition read only neutral texts, and subjects in the achievement priming condition were presented six achievement priming texts and 10 neutral texts (neutral texts were the same as in the neutral condition). Completed tasks were followed by no feedback, feedback regarding their performance compared to others (social reference norm; feedback indicating performance above or below the mean performance of others), or feedback regarding their performance compared to their performance in the task before (individual reference norm). Positive (indicated by a plus sign) and negative feedback (indicated by a minus sign) was equally varied within subjects. Independent of their performance, all subjects randomly received eight positive and eight negative feedback notifications. After completion of the experimental session, an explicit achievement motive questionnaire and other questionnaires not relevant for the present analyses were administered. To control for the processing of the priming texts, a recognition task was administered. Specifically, we selected one sentence from each text as well as sentences that had not been previously presented. The number of correctly classified sentences (whether they had been presented before or not) served as a measure of processing depth of the texts. We then tested subjects’ awareness of the experimental manipulation. We first asked what they thought the experiment was about and, in a second more specific question, if they thought that the text influenced their behavior in the task. Finally, subjects indicated their age and gender.

#### Priming Material 1

Excerpts taken from 9^th^ grade math textbooks served as the basis of our materials. In math books, longer passages of text are found at the beginning of new topics such as texts illustrating the relevance of problems in everyday life or the introduction of the mathematician who first solved a mathematical problem. Additionally, passages of text are also used to introduce or exemplify mathematical problems or in task descriptions. We looked for textual passages with clear achievement or neutral content. Achievement content was assessed on the basis of Winter’s [37] scoring manual for running text. The manual has been validated and has shown extensive predictive validity for various applications [38–41]. A text is classified as containing achievement imagery when a concern with a standard of excellence is expressed, such as doing good, reaching an achievement goal, winning or competing, positive and negative evaluation of success and failure, and unique accomplishments. The first author selected the passages for the experiment. He reached more than 90% agreement with Winter’s [37] manual for achievement imagery. Some textual passages were slightly modified to make the achievement content more explicit and prevalent or to make the text clearly neutral. The lengths of the texts varied between 36 and 79 words with *M* = 57.23 (*SD* = 10.53) (*M* = 55.00 for neutral and *M* = 60.80 for achievement texts). In total, we had 16 neutral and 10 achievement texts and, within the priming conditions, texts were selected randomly. Examples of the passages with clear achievement content for the achievement texts are: “Upon returning to Paris, he amazed his former teachers LaPlace and LaGrange with the most recent insights in geometry; The world record in typing the fastest SMS message; With my experience and concentration, I am getting better at doing this work.” The neutral text excerpts did not contain any achievement content according to the coding system in Winter’s manual. All texts are listed in Table A (English) and Table B (German) in S1 Priming Material.

#### Arithmetic Task 1

We used the arithmetic tasks from the “Concentration Performance Test” [42]. The tasks are highly standardized, with time per task and difficulty being the same for each block. The tasks consist of simple arithmetic problems with an additional rule. Specifically, subjects are presented two lines of three single numbers. Subjects have to add the two lines of numbers separately, and if the sum of the first line is larger than the second, the sums of the second line has to be subtracted from the first. If the sum of the first line is lower than the second, the two sums have to be added together. After the result is entered, the next two lines of numbers appeared on the screen.

### Results 1

Data were subjected to an analysis of variance (ANCOVA) with the dependent variable being the mean number of correctly solved arithmetic tasks per trial. We included baseline performance as a covariate to control for the ability of the subjects, the two experimental between-subjects conditions (priming and feedback), and the interactions of the experimental conditions. We also probed for the effects of age, gender, and processing depth (included as factors) in additional analyses and present the results for these variables first.

We found no main effect or interaction effect for age on performance (*p*s > .191). We found that men solved more tasks than women; *F*(1, 146) = 4.16, *p* = .043, *η*_*p*_^*2*^ = .028. Men solved *M* = 6.02 (*SE* = 0.17) and women *M* = 5.61 (*SE* = 0.11) tasks (men already solved more tasks in the baseline measure; *t*(157) = 4.00, *p* < .001; *M* = 5.69 (*SE* = 0.32) vs. *M* = 4.37 (*SE* = 0.17)). Beside the main effect, there were no significant interaction effects of gender and the experimental conditions on performance (*p*s > .140), and including gender did not substantially alter the effects of the conditions. On a descriptive level, priming effects were stronger for men. The mean difference for men between the neutral condition and the priming condition was *M*_*diff*_ = 0.75, and for women *M*_*diff*_ = 0.29; *F*(2, 146) = 1.45, *p* = .231, *η*_*p*_^*2*^ = .010. Regarding the three-way-interaction, priming effects were found across all feedback conditions for men whereas for women priming effects were found for the no feedback condition; *F*(2, 146) = 0.98, *p* = .379, *η*_*p*_^*2*^ = .013. Taken together, we did not include age and gender in our main analyses. Analyses with performance in the recognition tasks revealed no main effects and interaction effects (*p*s > .762). Thus, processing depth is of no importance (assuming all subjects actually read the text).

Table 1 presents the estimated means (ANCOVA) of performance in the experimental conditions. The ANCOVA revealed a main effect of baseline performance; *F*(1, 152) = 464.01, *p* < .001, *η*_*p*_^*2*^ = .753. Thus, about 75% of the performance variance in the experimental task was explained by the baseline performance. There was a significant main effect for priming; *F*(1, 152) = 7.32, *p* = .008, *η*_*p*_^*2*^ = .046. Subjects in the neutral condition solved *M* = 5.50 (*SE* = 0.12) tasks compared to *M* = 5.98 (*SE* = 0.13) in the achievement priming condition. Thus, our expectation that the achievement priming fosters performance was supported. The effect was rather small as it explained less than 5% of the variance in performance. There was a main effect for feedback; *F*(2, 152) = 4.72, *p* = .010, *η*_*p*_^*2*^ = .058. Post hoc comparisons (Bonferroni corrected) revealed that subjects in the feedback condition with an individual reference norm (*M* = 6.11, *SE* = 0.15) solved more tasks than in the no feedback condition (*M* = 5.51, *SE* = 0.16; *p* = .017) and, on a marginally significant level, more than in the social reference norm condition (*M* = 5.61, *SE* = 0.15; *p* = .053). The no feedback and the social reference norm condition did not differ substantially (*p* = .959). The interaction between prime and feedback condition was not significant; *F*(2, 152) = 1.60, *p* = .205, *η*_*p*_^*2*^ = .021. Thus, we could not support our expectation that priming effects are stronger with feedback.

**Table 1:** Estimated means (standard errors) of performance as a function of experimental conditions (Experiments 1).

		Feedback		
		No	Social RN^a^	Individual RN^a^
Prime	neutral	5.33 (0.22)	5.15 (0.23)^b^	6.02 (0.20)
	achievement	5.67 (0.23)	6.06 (0.20)^b^	6.20 (0.22)

*N* = 159

^a^ RN = Reference Norm

^b^ Means significantly differ (see post hoc ANCOVA described in text)

**Fig 1:**
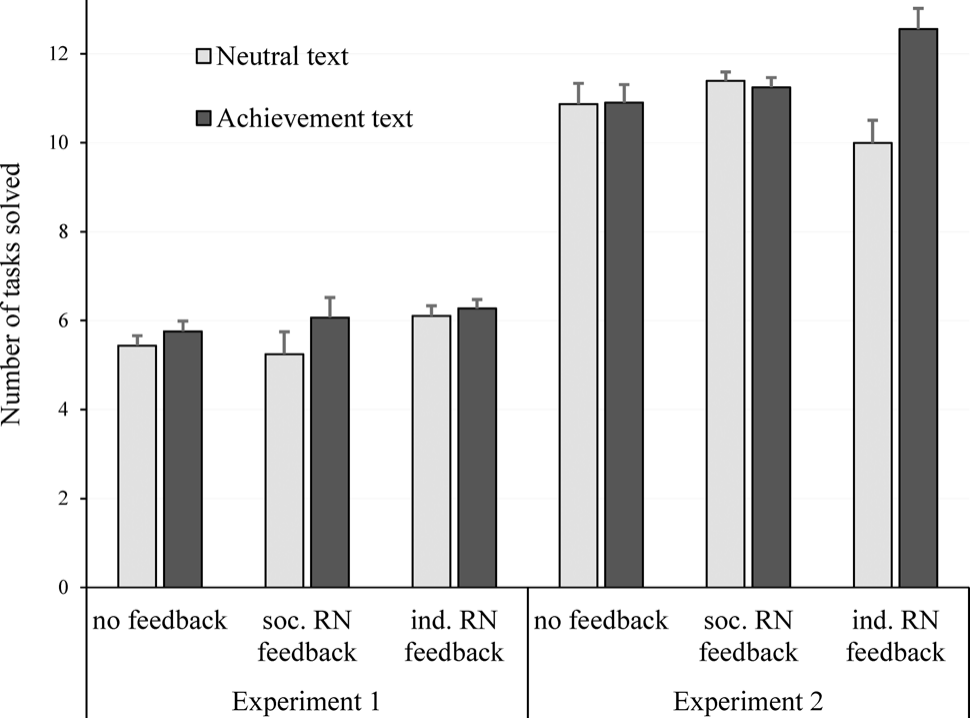
Estimated performance and standard errors as a function of baseline performance, achievement priming, and feedback (RN = Reference Norm; Experiments 1 and 2).

Fig 1 (left hand side) provides a graphical depiction of estimated means and standard errors of the ANCOVA just reported. The effect of priming is clearly visible, and it shows that achievement was higher in the feedback condition with an individual reference norm. Although there was no statistically significant interaction effect of priming and feedback, the priming effect was more pronounced in the social reference norm condition. A separate post hoc ANCOVA revealed a statistically substantial priming effect for this condition (*p* = .010, *η*_*p*_^*2*^ = .126). In the no feedback condition and in the individual reference norm condition, the effects were not statistically significant; *p* = .332, *η*_*p*_^*2*^ = .021, and *p* = .611, *η*_*p*_^*2*^ = .005), respectively. Therefore, we found some evidence that the priming effect was qualified by a stronger priming effect in the *social* reference norm feedback condition.

### Discussion 1

Semantic achievement primes in excerpts of mathematics schoolbooks showed a reliable albeit small priming effect on performance in an arithmetic task. This implies that behavioral priming effects found for single words or short statements generalize to this kind of stimuli. This also implies that semantic achievement priming could be of substantial relevance in educational settings. We could not support our expectations that feedback moderated the priming effect, but found some evidence that priming effects are especially strong in the feedback condition with a *social* reference norm.

Before taking this into consideration theoretically, we wanted to replicate the priming effect for running text in another main topic of school achievement: language. We also included high school students in our sample. Other aspects of our second experiment are essentially the same as in Experiment 1. We expect the priming effect of achievement primes in running text will be supported for this domain, too. We again tested the expected moderation effect of feedback as in Experiment 1.

## Experiment 2

### Method 2

#### Subjects and Design 2

In total, 182 subjects took part in our second study which was conducted at a University in Germany. 96 subjects were university students and 86 were high school students. Five subjects encountered software problems or they stopped working on the task, and seven subjects responded nonsensically on the anagram task. These 12 subjects were all university students and were excluded from the analysis. Eight subjects (four in each sample) did not indicate their age and gender, and we replaced the missing values by the population mean (for age within the subsamples). None of the subjects were aware of the experimental manipulations. The ages of the 84 university students (12 majoring in psychology) ranged from 18 to 32 years (*M* = 22.95, *SD* = 2.68), and 55 participants were women. The 86 high school students’ age ranged from 14 to 18 years (*M* = 15.43, *SD* = 0.95), and 49 subjects were women. The high school students attended local schools, and all subjects were paid for participation (15 € for university and 12 € for high school students). The information of participants and consent was the same as in Experiment 1, with the exception that high school students needed the additional written consent of the parents allowing their child to take part in the experiment.

We employed a 2 (neutral vs. achievement prime text content) x 3 (no feedback vs. social reference norm vs. individual reference norm) experimental between-subjects design. Performance was repeatedly measured in 10 trials.

#### Procedure 2

The procedure closely resembles that of Experiment 1. There are, however, two differences. First, we reduced the number of blocks to 10 (as we included high school students). Second, in the achievement priming condition, half of the texts were achievement priming texts (5 out of 10 instead of 6 out of 16 as in Experiment 1).

#### Priming Material 2

The priming material consisted of excerpts taken from 9^th^ grade schoolbooks. The length of the texts varied from 44 to 80 words (*M* = 57.56, *SD* = 10.14; *M* = 55.20 for neutral and *M* = 61.50 for achievement texts). Texts intended to prime achievement were selected in the same way as described in Experiment 1. Two of the priming texts were taken from Experiment 1 and four more were selected from 9^th^ grade language arts textbooks. In total, we had 10 neutral and six achievement texts, and within the priming conditions, texts were selected randomly. Examples of the passages with a clear achievement content for the achievement condition are: “I want to reach the top of the highest mountain in the world, and I want to be the first Danish woman to reach the summit of Mt. Everest; At the end of practice is expertise.” Five of the 10 neutral texts were taken from Experiment 1, and five were selected from 9^th^ grade language arts textbooks. The neutral text excerpts did not contain any achievement content according to the coding system in Winter’s manual. All texts are listed in Table A (English) and Table B (German) in S1 Priming Material.

#### Anagram Task 2

Anagrams, jumbled words containing five or six letters to be arranged into meaningful nouns, were tested in a pilot study. Overall, 500 anagrams were tested. Of these, 60 anagrams were taken from Voss [43] and 24 from a 9^th^ grade language arts textbook [44]. All others were self-generated. Eighty-four subjects took part in the pilot study. The anagrams were presented in 10 blocks of 20 anagrams. The blocks of anagrams were randomly selected, and each subject worked on 10 blocks. They had a maximum of 20 seconds to solve the anagram before the next one was presented. For the experimental task, we selected anagrams that were solved by at least 80% of the subjects, thus the anagrams selected for the experiment were rather easy. We excluded anagrams related to achievement. We also excluded anagrams with a strong positive or negative connotation, like joy (Freude) or anxiety (Angst). Anagrams in the experimental task were presented in randomly selected blocks of 22 anagrams with homogenous difficulty levels. Subjects were asked to solve as many anagrams as possible within 90 s. Unexpectedly, in 5% of the trials subjects completed more than the maximum of 22 anagrams selected for each block, in which case the list for that trial started over from the beginning. This resulted in a *M* = 1.10 s advantage when subjects solved the anagram for the second time. We created a formula to correct for this time advantage and conducted the analyses with the corrected scores and the original scores. No substantial differences in the observed effects were found. The same holds true when we replaced values higher than 22 with 22. This led us to use the original data without the corrected scores for the results presented here.

### Results 2

The analyses were executed analogous to Experiment 1. We found no main effect or interaction effect for age on performance (*p*s > .133) as well as for gender (*p*s > .280). On a descriptive level, priming effects were—in contrast to Experiment 1—weaker for men than women. The mean difference for men between the neutral condition and the priming condition was *M*_*diff*_ = 0.32, and for women *M*_*diff*_ = 1.21; *F*(2, 157) = 1.17, *p* =, 280, *η*_*p*_^*2*^ = .007. As there were no statistically reliable effects, we did not include these variables in our main analyses. Analyses with performance in the recognition tasks revealed no main effects and two-way interaction effects (*p*s > .137). Only the three-way interaction revealed a marginally significant interaction (*p* = .068) with achievement priming effects for social reference feedback condition decreasing the higher the performance in the recognition tasks was.

Table 2 presents the estimated means and SEs (ANCOVA) of performance in the experimental conditions. The ANCOVA revealed a main effect of baseline performance; *F*(1, 163) = 445.92, *p* < .001, *η*_*p*_^*2*^ = .732. Thus, about 73% of the performance variance in the experimental task was explained by the baseline performance. There was a significant main effect for priming; *F*(1, 163) = 4.56, *p* = .034, *η*_*p*_^*2*^ = .027. Subjects in the neutral condition solved *M* = 10.75 (*SE* = 0.29) tasks compared to *M* = 11.57 (*SE* = 0.25) in the achievement priming condition. Thus, our expectation that achievement priming fosters performance was again supported. The effect was rather small and explained less than 3% of the variance in performance. There was no main effect for feedback; *F*(2, 163) = 0.52, *p* = .598, *η*_*p*_^*2*^ = .006. The interaction between prime and feedback condition was significant; *F*(2, 163) = 5.03, *p* = .008, *η*_*p*_^*2*^ = .058.

**Table 2:** Estimated means (standard errors) of performance as a function of experimental conditions (Experiments 2).

		Feedback		
		No	Social RN^a^	Individual RN^a^
Prime	neutral	10.87 (0.50)	11.39 (0.47)	9.99 (0.51)^b^
	achievement	10.90 (0.45)	11.25 (0.41)	12.55 (0.47)^b^

*N* = 170

^a^ RN = Reference Norm

^b^ Means significantly differ (see post hoc ANCOVA described in text)

Separate post hoc analysis for each feedback condition showed no priming effect for the no feedback condition (*p* = .946, *η*_*p*_^*2*^ < .001), no priming effect for the social reference norm condition (*p* = .742, *η*_*p*_^*2*^ = .002), but a substantial priming effect in the individual reference norm condition (*p* < .001, *η*_*p*_^*2*^ = .236). Thus, our expectation that the priming effect will be stronger in both feedback conditions could not be supported. Only in the individual reference norm condition did achievement priming lead to higher performance. Fig 1 (right hand side) provides a graphical depiction of estimated means just reported. The means showed that the main effect of priming was qualified by a strong effect in the individual reference norm condition (and not in the other two feedback conditions).

Including the sample as an additional factor revealed a main effect for sample; *F*(1, 157) 13.25, *p* < .001, *η*_*p*_^*2*^ = .078. University students solved *M* = 11.90 (*SE* = 0.29) words and high school students *M* = 10.48 (*SE* = 0.26). The effects for experimental conditions changed in the way that the main effect for priming was less strong and only close to being statistically significant; *F*(1, 157) = 3.85, *p* = .051, *η*_*p*_^*2*^ = .024. The main effect for feedback did not change substantially; *F*(2, 157) = .665, *p* = .515, *η*_*p*_^*2*^ = .008. The two-way interaction of prime and feedback was also weaker when sample was included and only marginally significant; *F*(1, 157) = 2.60, *p* = .078, *η*_*p*_^*2*^ = .032. The two-way interactions of sample with the experimental conditions were not statistically significant (*p* = .780 for prime and *p* = .853 for feedback conditions), but the three-way interaction revealed a marginally significant effect; *F*(1, 157) = 2.55, *p* = .082, *η*_*p*_^*2*^ = .031. Inspection of the means revealed that priming effects for the high school students were found in both conditions with feedback (more pronounced for the social reference norm), but not in the no feedback condition. For university students, priming effects were found for the no feedback condition and in the individual reference norm feedback condition, but not for the social reference norm feedback condition. Thus, on a descriptive level, priming effects for the individual reference norm feedback condition were found in both samples whereas for the other conditions the effects were not in the same direction.

### Discussion 2

We found support for our expectations as semantic achievement primes of excerpts of schoolbooks fostered performance in the language domain. This implies that behavioral priming effects found for single words or short statements generalize to this kind of stimuli in the language domain as well. We could not support our expectations that feedback per se moderated the priming effect. However, the priming effect was stronger in the feedback condition with an individual reference norm, thus indicating that the type of feedback did in fact influence priming effects. Post hoc analysis with sample as an additional factor revealed a marginally significant three-way interaction giving some evidence that the priming effect for the individual reference norm condition was found for both samples while for the no feedback and social reference norm conditions, priming effects were not in the same direction.

## General Discussion

We consistently found a semantic behavioral achievement priming effect of excerpts of schoolbooks in two domains: Achievement primes increased performance in an arithmetic task in Experiment 1 and in an anagram task in Experiment 2. In line with our reasoning, this implies that behavioral achievement priming generalizes to this kind of stimuli. The effects were rather small in both experiments and only the high power due to the high number of subjects enabled us to reliably detect the effect. Unexpectedly, the priming effects were independent of contextual factors in the first experiment with some evidence from post hoc analyses that the priming effect was stronger for the social reference norm feedback. In Experiment 2, priming effects were more pronounced when subjects received feedback with an individual reference norm.

The effect of achievement priming was found for two different tasks. In the arithmetic tasks in Experiment 1, subjects have to apply a different rule depending on the sums of the two lines. This task requires high concentration and substantial conscious volitional effort to succeed [45]. In contrast, the anagram task does not require this kind of volitional effort. Subjects have to identify the words and do not have to apply a specific additional rule imposed by the task. Therefore, our results not only allow for generalization of priming effects for a new method of achievement priming (excerpts from schoolbooks), but also for different kinds of tasks. This is especially important because in previous priming studies mainly creativity tasks such as finding words, creating new words or multiple uses of common objects have been used (cf. [4–8, 29]) with only few exceptions [46, 47].

In both experiments, the main effects of semantic achievement primes on performance show that priming effects generalize to achievement primes presented in running text and are not limited to single prime words. This implies that priming effects could be of considerable importance in natural contexts as well. More specifically, our data provide evidence that schoolbooks have achievement priming effects on students in the classroom. Correlational analyses support this reasoning [11]. Based on this previous (correlational) finding and our present results, it is reasonable to assume that each time students encounter achievement primes in textbooks they subsequently perform a little better. If this effect happens more frequently, this should cumulate to higher general self-confidence (cf. [21]) and higher ability (because students are assumed to be more focused on the task which should promote ability). Subsequently, this should result in better school performance in general regardless of whether achievement primes are absent or present in testing situations. However, intervention studies varying achievement imagery in the normal school context should be conducted in order to ascertain if an enrichment of achievement imagery indeed could be one element to foster performance of students. This is especially important as we found some evidence that context factors moderate priming effects (discussed below).

We anticipated a small priming effect and obtained a relatively large sample size in order to reliably detect the effect. If we would have sampled half the subjects—which would still be a common sample size (this would mean roughly 40 subjects in the neutral and achievement priming condition in the first and second experiment)—we would not have been able to conceptually replicate the priming effect with achievement primes in running text. However, sample size is only one aspect of the problem in replication in general and the replication of semantic behavioral priming effects in particular. The failed replication of the achievement priming effect of Bargh et al. [4] by Harries et al. [23] found no priming effect even on a descriptive level. One would have expected at least a small effect, if sample size had been the principle problem in the replication. We could only speculate why they failed to replicate the effect (when we were able to replicate it here).

One aspect may be that psychology students were included in their sample (which we almost successfully were able to avoid). Priming studies and their methodology are likely to be known by psychology students, which could have been influential even if subjects did not know the exact experimental manipulation. According to the situated inference model [18], priming effects occur only if subjects misattribute the primed information as an internal thought process. In a similar vein, achievement primes in running text may be less obvious or blur the experimental manipulation. We also repeatedly exposed subjects to achievement primes in several trials, but not in all trials. To repeat achievement priming may also have helped to detect the effect due to increased reliability of repeated priming (cf. [7]). Furthermore, we controlled for baseline performance. Finally, and as mentioned in the introduction, a more complete understanding of the behavior priming effect seems of central importance [16] in order to replicate the effect. We found a main effect for priming, but the main effect was (partly) qualified by context factors (i.e., feedback condition). Thus, the priming effect seems very sensitive to possibly even subtle context factors (cf. [7]), which makes replication likely to fail.

### The role of context

The context factor feedback moderated the priming effect to some extent. This stands in contrast with other research that found a distinct moderating influence of context factors (cf. [5, 7, 27, 29]). In Experiment 1, the priming effect was observed across all experimental conditions (with some evidence that it was stronger for the social reference norm condition). In retrospect, we would claim that the arithmetic task itself was sufficiently achievement related so that there was no need for further enhancement of the achievement character of the task. If the task had been less achievement related, the context would have possibly moderated the priming in line with our expectations. In contrast to Experiment 1, however, the priming effect in Experiment 2 was moderated by the reference norm of the feedback. The priming effect was stronger for the individual reference norm. On a descriptive level, there was no such moderation effect in Experiment 1, and the priming effect was even stronger for the social (and not for the individual) reference norm condition.

As the designs of the experiments were analogous, the different results between both experiments can be reasonably attributed to the different tasks. Although not assessed systematically, subjects’ verbal comments after the experiment indicated that they enjoyed the arithmetic tasks in Experiment 1 less than the anagram tasks in Experiment 2. When one enjoys a task and is primed to achieve, an individual reference norm may resemble a more natural context to execute achievement-related behavior. A social reference norm may only be needed if the task itself is less enjoyable (less intrinsically rewarding). These would be in line with results found by van Yperen [48] that the interplay of task interest and goals in respect to social and individual reference norms predicted performance (cf. [49]). For example, subjects who pursued goals with an individual reference norm (i.e., focused on individual progress) performed better when having high interest in the task, resembling the pattern we found for Experiment 2 (priming and individual reference norm feedback works for interesting tasks). In future research, it would be informative to test such a matching hypothesis between task qualities and context conditions on the achievement priming effect more systematically. We would expect a benefit of achievement priming for enjoyable tasks when feedback provides information about performance compared to one’s own past performance (i.e., individual reference norm). On a more general level, it can be assumed that the influence of external task contexts is not the same for all kinds of tasks (this includes that the task itself provides a context, which amplifies or compensates for task external contexts). This means, the effect of context differs according to task characteristics.

## Conclusion

Taken together, we provide evidence that achievement primes in running text foster performance. As the primes were taken from excerpts of school textbooks, it is reasonable to assume that priming effects are likely to happen in the classroom or in other natural settings as well. The context moderated the priming effect to a lesser extent than we expected. We highlight the necessity to further elaborate on the correspondence of prime and context in order to get a more conclusive empirical base in which situations primes do actually foster performance.

## Supporting Information

S1 Dataset Data from Experiment 1. (XLSX)

S2 Dataset Data from Experiment 2. (XLSX)

S1 Priming Material (DOC)
