# Supplementary material for: Schoolbook Texts: Behavioral Achievement Priming in Math and Language
Source: PLoS One. 2016 Mar 3;11(3):e0150497. doi: 10.1371/journal.pone.0150497 (PMC4777530; doi:10.1371/journal.pone.0150497)
Supplement: S1 Priming Material — (DOC) [file pone.0150497.s003.doc]

**S1 Priming Material**

**Table A. Priming material (English translations) for Experiment 1 and 2 (achievement imagery underlined)**

| **Achievement Texts** |
| --- |
| The world record in typing the fastest SMS text message is 41.52 seconds for 160 characters. This was accomplished by the then 16-year-old Ang Chuang Yang from Singapore. He typed more than three characters per second. How fast are you? Concentrate and type as fast as you can. (Experiments 1 and 2) |
| Elisa is her 2nd apprenticeship year as a pastry chef. Elisa said: "In the first year of training, I learned all about different doughs. Now in my 2nd year I am also learning how to make gingerbread. In the bakery/confectionery where I work, there is also a small café. I enjoy serving the customers and seeing how great my chocolates and cakes taste." (Experiments 1 and 2) |
| For his final examination at the "Ecole Militaire" in Paris, Napoleon (1769-1821) was tested by LaPlace. Thanks to his brilliant results, he was commissioned as an officer at the age of 16 years. During the military campaign in Italy (1796-1797), as commander in chief he sought contact to Italian mathematicians. Upon returning to Paris, he amazed his former teachers LaPlace and LaGrange with the most recent insights in geometry. (Experiment 1) |
| Deniz wants to be a mason. He talks about his apprenticeship: "Before, I didn’t think that it is so difficult to build a straight wall. I use the level to check if I'm still working correctly. On the construction site I often need a "mason‘s triangle," which is a string with knots. Using this, and with my experience and concentration, I am getting better at doing this work. "(Experiment 1) |
| Applicants for apprenticeships are often tested in writing before they are invited for an interview. This is done by occupational entrance exams. These tests are often very different in their form and content. Proficiencies can also be questioned that has not been covered in school. However, math problems from diverse areas are almost always dealt with. Prepare yourself well and concentrate in order to demonstrate your skill level in the best possible way. (Experiment 1) |
| For many problems you need a lot of patience and a good overview because the line of reasoning consists of several steps. You need strategies to find the necessary building blocks for the argument. During forward operations all the prerequisites and consequences are analyzed. One begins at the base of the argument. With backwards operations, you begin, somewhat unusually, "on the roof." You gather the conditions, from which the finding directly - in any case faster than taking the given conditions - results. (Experiment 1) |
| A building materials company has two brick production plants in which exclusive bricks can also be produced. The plant in Yanten can produce 24 square meters daily, the factory in Yburg 60 square meters daily. A customer places an order for more than 300 square meters of brick with the company and calculated: "If both plants are working on this order, it should be possible to complete it within 3.5 days." The production manager and the customer talk about how to find best solution for quickly finishing the job. (Experiment 1) |
| The approximation pi = 3 for the mathematical constant corresponds to the ancient Babylonian value for pi. Around 2000 B.C., the Egyptians were already aware of a very accurate calculation of the surface area of the circle. With the value pi = 3.16, a good approximation of pi was found 4,000 years ago, which differs by less than 1% in comparison to the correct value. An outstanding achievement. (Experiment 1) |
| The Zugspitze Mountain Railway is the longest cable railway in Germany. It leads from the valley station at the Eibsee to the summit of the Zugspitze. In July 1926, the first line of the Zugspitzbahn was opened, however, from the Austrian side. The Tyroleans were faster and thus won the race against the Bavarians for the technical development of the summit. (Experiment 1) |
| Gert Mittring is faster than any calculator and can determine the square root of the most incredible numbers. In such cases, even high-performance computers have capitulated. Calculating the 9,875 root of a 39,413 digit number is no problem for the Stuttgart native who resides in Bonn. After a maximum of 40 seconds, he presented the correct solution. (Experiment 1) |
| What matters during cursory reading is to grasp the essentials of content, that is, the most important information, quickly and thoroughly. Details or incidentals are disregarded. If one recognizes a point in a text or a book that is important for the solution of a problem, this should be read thoroughly and marked for later, careful study. (Experiment 2) |
| Christof reported: There is a lot of electronics in modern cars. I have to constantly better myself. You also cannot be afraid of getting your hands dirty. As a team we work hand in hand, often under time pressure. Nevertheless, we have to work reliably and accurately. Because the most important thing is that the customer gets a safe car. I am also responsible for this. (Experiment 2) |
| Practice is an exhausting affair. It's like a workout in sports, which is also a kind of practice: It takes sweat and perseverance. Sheer pleasure it is not. And that’s how it is when practicing vocabulary, learning a poem, or drafting a text. What's fun is when you have learned something after lots of practice. At the end of practice is the expertise. And being able to do something is satisfying. (Experiment 2) |
| I want to go to the top of the highest mountain in the world. I want to be the first Danish women to reach the summit of Mount Everest. I know that I'm experienced enough to climb the next ledge, know that only my old fears can stop me, know that I have the intellectual resources to overcome them. I want to be on the top - at any price - so I rise up and begin with the crossing on the way to the summit. (Experiment 2) |
| **Neutral Texts** |
| Neustadt. Some like it, others don’t. However, the conclusion was clear, even if the topic was discussed for hours. With a majority of votes, the local council in Neustadt has agreed to the construction of two 1.5-megawatt wind turbines. Have a debate about the pros and cons of wind turbines. (Experiments 1 and 2) |
| Felicitas hears some news at 9 clock. One minute later she confidentially shares this with her girlfriend. After another minute, each of them confidentially share this news with yet another classmate. Felicitas believes that by end of school all 1,000 students at her school will know the news. (Experiments 1 and 2) |
| A lot of children and teenagers get pocket money from their parents. For most 14- to 16-year-olds, the allowance amounts to about 20 to 25 € per month. Some save part of their pocket money and put it a checking or savings account. There, one can simply wait while the money earns interest. (Experiments 1 and 2) |
| On 26 April 1986, a severe nuclear accident occurred at the Chernobyl Nuclear Power Plant close to Kiev. Large quantities of radioactive substances were released in this design basis accident (DBA). Winds spread the substances across large parts of Europe. Because the radioactive substances were deposited on the soil and plants, they even entered the food chain. As they emit radiation, radioactive materials are transformed into other substances (they "decay"). (Experiments 1 and 2) |
| Janine talks about her training as a hairdresser: "I've always liked to help my girlfriends color their hair. Now I'm in my first year of training as a hairdresser at "Salon Curl", and I have given models haircuts and perms Most customers are friendly, and I like to talk to them. But sometimes I find it hard to be standing up almost the entire day." (Experiments 1 and 2) |
| Sina is training to be a sales associate: "I'm doing an apprenticeship at a large clothing chain. This way I can get to know many areas. I accept the deliveries of goods, sort the merchandise into the racks, and price the goods. At the sales counter I check whether the banknotes are genuine." (Experiment 1) |
| Sibylle has chosen to do an apprenticeship as a painter and varnisher. "I often go to homes or offices with the other apprentices and the master craftsman and paint there according to the specifications of the customer. It's fun to work with my colleagues. In vocational school I'm with the other apprentices, and otherwise we do a lot together, too." (Experiment 1) |
| When a ship travels upstream, its speed compared to the bank is the difference between the speed of the ship in calm water and the flow velocity of the flowing water. When traveling downstream, however, the two speeds are added. The motor vessels of the Swiss Shipping Company will need 35 minutes for the 10 km route in the part of the Rhine River known as the High Rhine from Stein am Rhein to Diessenhofen, and traveling in the opposite direction they will require 60 minutes. (Experiment 1) |
| Grandfather Brinkmann presents each of his grandchildren, Karin, Inga, and Lars, a savings account. He would like each child to receive the same amount. Grandmother Balzer has a balance of 16,912.00 Euros in her savings account. She would like to give her seven grandchildren half of that amount. (Experiment 1) |
| There are cans that are tall and thin or flat and wide. Drinks are usually poured into thinner cans, while fruits and vegetables tend to be stored in wider cans, and sausage products are often in flat containers. Therefore, one can associate certain goods to specific can shapes. (Experiment 1) |
| Silhouettes of people have been found in Palaeolithic cave paintings, in Egyptian art, and in Greek vase painting. In a silhouette portrait of a person, the outline of the head or the whole body was traced on a contrasting colored background. The more common usage of silhouette can be traced back to King Louis XV’s minister of finance, Etienne de Silhouette (1709-1767). He decreed nationwide austerity measures, thereby contributing to the proliferation of the silhouette compared to the more expensive portrait. (Experiment 1) |
| In the past, gas in Oberhausen was stored in the Gasometer. Floating on the surface was a gas pressure disc that provided for the necessary gas pressure. Nowadays, though, it is securely attached, and at the height of 4.5 meters, is the platform of the Gasometer. Since 1994, the Gasometer is used as an exhibition and event venue. Here, people can relax and recover from the daily grind. (Experiment 1) |
| On its website the environmental group Greenpeace declared: "Lignite is a problematic source of energy. For mining, the ground water is extensively lowered, people are driven out of their villages, and the fine dust contaminates their lungs. The large amounts of greenhouse gas emissions set free during the combustion of coal contribute to climate change. "Discuss the pros and cons of coal power plants. (Experiment 1) |
| An oil tanker lost a large amount of oil. The coast was polluted by the oil spill. Many people are engaged in the cleanup, yet the cleaning up process takes a long time and the contamination is still present after many years. The feathers of birds are affected, and many other animals die from asphyxiation and poisoning. (Experiment 1) |
| To play the lottery a person chooses six numbers from the numbers 1 to 49. At the drawing, balls labeled with the numbers 1 to 49 are put into the lottery wheel. One after the other, six numbers and a bonus number are drawn. In order have “all six numbers” you need a lot of luck. The government is the highest lottery earner, and this most of all through the levied taxes and duties from the lottery. (Experiment 1) |
| In certain television shows, viewers are encouraged to call as often as possible for a particular candidate or to answer easy questions. In most cases the caller does not get through. There is only a recording that can be heard: "... Hello! Thank you for calling. Unfortunately, you were not lucky this time. Feel free to try your luck again. This call will cost you 50 cents." (Experiment 1) |
| Every Monday, Tuesday, and Thursday morning they rode together to school. If he was able to keep the seat next to him free until her bus stop, they sat next to each other. In the crowded bus they shared their own deserted island: A seated island or a standing island, where they talked and talked and talked. (Experiment 2) |
| The training as a professional makeup artist is not uniformly regulated nationwide: there may be differences from company to company and from school to school. Currently, you can study the profession in theaters, television stations, and private schools. The training lasts two to four years. Often, a completed hairdressing apprenticeship is a prerequisite. (Experiment 2) |
| Achim slid halfway out of the bed. Five past one. Again too late. He stared at the ceiling. Know. Nothing. A blank sheet of paper, an unpainted picture, a soundless melody, an unspoken word, unlived life. Half body turn to the right, a touch on the power button of his system. Sometimes music raised him up. (Experiment 2) |
| Livestock production, which now is verging on madness, demands a high price not only from the fattened animals, but also from the environment. Soil and water are poisoned by the enormous quantities of liquid manure, resulting from the high animal density in breeding establishments. Nitrogen compounds pass via streams and rivers into the oceans and lakes and are responsible for the increased growth of algae. (Experiment 2) |
| Dialects are local or regional special forms of a language. Tracing back to the Latin 'dialectos" (= expression), with “Mundarten,” Philip of Zesen Germanized the term in the 17th century. Dialects belong to the language varieties. Linguists use the term to avoid possible prejudice against dialects. In principle, mutual identification is preserved in the dialects of a language. Nonetheless, there can be very large deviations in pronunciation, vocabulary, and grammar as well as in the meaning and use of words. (Experiment 2) |

**Table B. Priming material for Experiment 1 and 2 (achievement imagery underlined)**

| **Achievement Texts** |
| --- |
| Der Weltrekord im SMS-Schnelltippen liegt bei 41,52 Sekunden für 160 Zeichen. Das hat der damals 16-jährige Ang Chuang Yang aus Singapur geschafft. Pro Sekunde hat er also mehr als drei Zeichen getippt. Wie schnell bist du? Konzentriere dich und tippe so schnell du kannst. (Experiment 1 and 2) |
| Elisa ist im 2. Lehrjahr als Konditorin. Elisa erzählt: „Im 1. Ausbildungsjahr habe ich alles über verschiedene Teige gelernt. Jetzt im 2. Jahr lerne ich auch Lebkuchen herzustellen. In der Bäckerei und Konditorei, in der ich arbeite, gibt es auch ein kleines Café. Es macht mir Spaß, Kunden zu bedienen und zu sehen, wie gut ihnen meine Pralinen und Kuchen schmecken." (Experiment 1 and 2) |
| Napoleon (1769-1821) wurde bei der Abschlussprüfung an der „Ecole militaire“ in Paris von LaPlace geprüft. Dank eines glänzenden Ergebnisses wurde er schon mit 16 Jahren zum Offizier ernannt. Während des Feldzuges in Italien (1796-1797) suchte er als Oberbefehlshaber Kontakt zu italienischen Mathematikern. Bei der Rückkehr nach Paris verblüffte er seine früheren Lehrer LaPlace und LaGrange mit neuesten Erkenntnissen aus der Geometrie. (Experiment 1) |
| Deniz wird Maurer. Er erzählt von seiner Ausbildung: „Vorher hätte ich nicht gedacht, dass es so schwierig ist, eine gerade Mauer zu bauen. Mit der Wasserwaage messe ich nach, ob ich noch richtig arbeite. Auf dem Bau benötige ich oft ein „Maurerdreieck", das ist eine Schnur mit Knoten. Damit und durch meine Erfahrung und Aufmerksamkeit gelingt mir die Arbeit immer besser." (Experiment 1) |
| Bewerber für Ausbildungsplätze werden oft schriftlich getestet, bevor sie zu einem Gespräch eingeladen werden. Dies geschieht durch Berufseingangstests. Diese Tests sind in ihrer Form und ihrem Inhalt oft sehr unterschiedlich. Es können auch Kenntnisse abgefragt werden, die nicht in der Schule behandelt wurden. Es werden jedoch fast immer Mathematikaufgaben aus unterschiedlichen Gebieten behandelt. Bereite dich gut vor und konzentriere dich, um dein Können optimal zu zeigen. (Experiment 1) |
| Bei vielen Problemen benötigt man einen langen Atem und einen guten Überblick, weil die Argumentationskette sich aus mehreren Schritten zusammensetzt. Man benötigt Strategien, um die notwendigen Baustein für die Argumentationskette zu finden. Beim Vorwärtsarbeiten analysiert man alle Voraussetzungen und die Folgerungen. Man fängt sozusagen am Fundament des Argumentationsgebäudes an. Beim Rückwärtsarbeiten fängt man etwas ungewohnt „am Dach“ an. Man sammelt die Bedingungen, aus denen die Behauptung direkt, jedenfalls schneller als aus den gegebenen Voraussetzungen, folgen würde. (Experiment 1) |
| Eine Baustofffirma hat zwei Ziegelsteinwerke, in denen auch besondere Ziegelsteine hergestellt werden können. Das Werk in Yanten kann davon täglich 24 Quadratmeter produzieren, das Werk in Yburg täglich 60 Quadratmeter. Ein Kunde erteilt der Firma einen Auftrag über 300 Quadratmeter Ziegel und schätzt: „Wenn beide Werke daran arbeiten, müsste das doch innerhalb von 3,5 Tagen zu schaffen sein“. Der Produktionsleiter und der Auftraggeber unterhalten sich, wie hier die beste Lösung für das schnelle Erledigen der Aufträge gefunden werden kann. (Experiment 1) |
| Die Näherung Pi = 3 für die Kreiszahl entspricht dem altbabylonischen Wert für Pi. Die Ägypter kannten schon etwa 2000 Jahre vor Christus eine sehr genaue Berechnung des Flächeninhaltes des Kreises. Mit Pi = 3,16 wurde vor 4000 Jahren eine gute Näherung für Pi gefunden, die sich um weniger als 1% vom richtigen Wert unterscheidet. Eine herausragende Leistung. (Experiment 1) |
| Die Zugspitzbahn ist die längste Seilbahn in Deutschland. Sie führt von der Talstation am Eibsee auf den Gipfel der Zugspitze. Im Juli 1926 wurde die erste Zugspitzbahn eröffnet, jedoch von der österreichischen Seite aus. Die Tiroler waren schneller und gewannen damit den Wettlauf mit der bayerischen Seite um die technische Erschließung des Gipfels. (Experiment 1) |
| Gert Mittring ist schneller als jeder Taschenrechner und zieht die Wurzeln aus den unglaublichsten Zahlen. Da kapitulieren sogar Hochleistungscomputer. Die 9 875. Wurzel aus einer 39413-stelligen Zahl zu ziehen, ist kein Problem für den gebürtigen Stuttgarter mit Wohnsitz in Bonn. Nach maximal 40 Sekunden präsentierte er die richtige Lösung. (Experiment 1) |
| Beim kursorischen Lesen geht es darum, das inhaltlich Wesentliche, also die wichtigsten Informationen, schnell und gründlich zu erfassen. Einzelheiten oder Nebensächlichkeiten bleiben unberücksichtigt. Erkennt man in einem Text oder einem Buch eine Stelle, die für die Lösung einer Aufgabe wichtig ist, so liest man sie gründlich und kennzeichnet sie für ein späteres sorgfältiges Studieren. (Experiment 2) |
| Christof berichtet: In modernen Autos steckt viel Elektronik. Da muss ich mich ständig weiterbilden. Man darf auch keine Scheu haben, sich die Finger schmutzig zu machen. Im Team arbeiten wir Hand in Hand, häufig auch unter Zeitdruck. Trotzdem müssen wir zuverlässig und genau arbeiten. Denn das Wichtigste ist, dass der Kunde ein sicheres Auto bekommt. Dafür bin auch ich verantwortlich. (Experiment 2) |
| Üben ist eine anstrengende Sache. Es ist wie beim Training im Sport, das ebenfalls eine Art des Übens ist: Es kostet Schweiß und Durchhaltevermögen. Ein reines Vergnügen ist es nicht. So ist es auch, wenn man Vokabeln übt, ein Gedicht lernt oder einen Text erarbeitet. Was Spaß macht, ist, wenn man nach langem Üben etwas gelernt hat. Am Ende des Übens steh das Können. Und etwas zu können bereitet Vergnügen. (Experiment 2) |
| Ich will auf die Spitze des höchsten Berges der Welt. Ich will die erste Dänin sein, die den Gipfel des Mount Everest erreicht. Ich weiß, dass ich erfahren genug bin, um den nächsten Absatz zu erklettern, weiß, dass mich nur meine alten Ängste aufhalten können, weiß, dass ich die geistigen Ressourcen habe, um sie zu überwinden. Ich will an die Spitze - um jeden Preis -, also erhebe ich mich und beginne mit der Querung auf dem Weg zum Gipfel. (Experiment 2) |
| **Neutral texts** |
| Neustadt. Den einen gefällt es, den anderen nicht. Der Beschluss war jedoch eindeutig, auch wenn über das Thema über Stunden diskutiert wurde. Der Gemeinderat in Neustadt hat mit einer Stimmenmehrheit dem Bau von zwei 1,5-Megawatt-Windkraftanlagen zugestimmt. Führe ein Streitgespräch über Pro und Contra von Windkraftanlagen. (Experiment 1 and 2) |
| Felicitas erfährt um 9 Uhr eine Neuigkeit. Nach einer Minute erzählt sie diese ganz vertraulich einer Freundin weiter. Nach einer weiteren Minute erzählen beide wieder ganz vertraulich die Neuigkeiten jeweils einem weiteren Mitschüler. Felicitas glaubt, dass bis Schulschluss alle 1000 Schülerinnen und Schüler ihrer Schule die Neuigkeit kennen. (Experiment 1 and 2) |
| Viele Kinder und Jugendliche bekommen von ihren Eltern Taschengeld. Das Taschengeld beträgt bei den meisten 14- bis 16-jährigen Jugendlichen 20 bis 25€ im Monat. Manche Schüler sparen einen Teil ihres Taschengeldes und legen es auf einem Konto oder Sparbuch an. Dort bringt das Geld Zinsen und man kann in Ruhe abwarten. (Experiment 1 and 2) |
| Am 26. April 1986 ereignete sich im Atomkraftwerk Tschernobyl bei Kiew ein schwerer Reaktorunfall. Bei dem GAU wurden große Mengen radioaktiver Stoffe freigesetzt. Diese Stoffe wurden durch den Wind über große Teile Europas verbreitet. Durch Ablagerung auf Böden und Pflanzen gelangten diese Stoffe auch in die Nahrungskette. Radioaktive Stoffe wandeln sich unter Abgabe von Strahlung in andere Stoffe um (sie „zerfallen“). (Experiment 1 and 2) |
| Janine erzählt von ihrer Ausbildung zur Friseurin: "Ich habe immer gern meinen Freundinnen beim Haare färben geholfen. Jetzt bin ich im ersten Lehrjahr zur Friseurin im "Salon Locke" und habe Modellen die Haare geschnitten und Dauerwellen gemacht. Die meisten Kundinnen und Kunden sind freundlich und ich mag es, mit ihnen zu reden. Nur manchmal finde ich es anstrengend, fast den ganzen Tag zu stehen." (Experiment 1 and 2) |
| Sina macht eine Ausbildung zur Verkäuferin: "Ich mache meine Ausbildung bei einer großen Bekleidungskette. So kann ich in viele Bereiche kennen lernen. Ich nehme Warenlieferungen an, räume die Ware in Regale ein und zeichne die Preise aus. An der Kasse prüfe ich, ob die Geldscheine echt sind." (Experiment 1) |
| Sibylle hat sich für eine Ausbildung zur Malerin und Lackiererin entschieden. "Ich fahre oft mit den Gesellen und dem Meister in Wohnungen oder Büros und streiche dort nach den Vorgaben der Kunden. Es macht Spaß, mit meinen Kollegen zusammen zu arbeiten. lm Berufskolleg bin ich mit den anderen Lehrlingen zusammen und wir machen auch sonst viel zusammen." (Experiment 1) |
| Fährt ein Schiff einen Fluss bergwärts, so ist seine Geschwindigkeit gegenüber dem Ufer die Differenz der Eigengeschwindigkeit des Schiffes in ruhendem Wasser und der Strömungsgeschwindigkeit des fließenden Wassers. Bei der Talfahrt dagegen werden die beiden Geschwindigkeiten addiert. Die Motorschiffe der Schweizerischen Schifffahrtsgesellschaft benötigen für die 10km lange Strecke auf dem Hochrhein von Stein am Rhein nach Diessenhofen 35 Minuten und in umgekehrter Richtung 60 Minuten. (Experiment 1) |
| Opa Brinkmann schenkt seinen Enkelkindern Karin, Inga und Lars ein Sparbuch. Er möchte, dass jedes Kind den gleichen Betrag erhält. Oma Balzer hat auf ihrem Sparbuch ein Guthaben von 16912,00 Euro. Sie möchte die Hälfte des Betrages ihren sieben Enkeln schenken. (Experiment 1) |
| Es gibt hohe dünne und flache breite Dosen. Getränke werden meistens in dünnere Dosen abgefüllt, Früchte und Gemüse eher in breitere Dosen und Wurst oft in flachen Dosen. Man kann demnach bestimmten Waren bestimmte Dosenformen zuordnen. (Experiment 1) |
| Schattenrisse von Personen findet man schon in altsteinzeitlichen Höhlenmalereien, in der ägyptischen Kunst und in der griechischen Vasenmalerei. Für ein Schattenporträt einer Person wurden die Umrisse des Kopfes oder des ganzen Körpers auf einem kontrastfarbigen Untergrund nachgezeichnet. Der gebräuchlichere Begriff der Silhouette geht auf den Finanzminister Ludwigs des XV., Ètienne de Silhouette (1709-1767), zurück. Dieser verfügte Sparmaßnahmen des Staates und trug damit zur Verbreitung des Schattenrisses im Vergleich zum teureren Porträt bei. (Experiment 1) |
| Früher wurde in dem Gasometer in Oberhausen Gas aufbewahrt. Auf dem Gas schwamm eine heute noch vorhandene Scheibe, die für den notwendigen Gasdruck sorgte. Sie ist aber inzwischen befestigt und stellt die Plattform des Gasometers in 4,5 Metern Höhe dar. Seit 1994 wird der Gasometer als Ausstellungs- und Veranstaltungsort genutzt. So können sich die Leute dort ausruhen und vom Alltag erholen. (Experiment 1) |
| Die Umweltschutzorganisation Greenpeace erklärt auf ihrer Webseite: "Braunkohle ist ein problematischer Energieträger. Für den Abbau wird großflächig das Grundwasser abgesenkt, Menschen werden aus ihren Dörfern vertrieben, und der Feinstaub belastet die Lungen der Menschen. Die großen Mengen der bei der Verbrennung von Kohle frei gesetzten Treibhausgase tragen zum Klimawandel bei." Diskutiere das Pro und Contra von Kohlekraftanlagen. (Experiment 1) |
| Ein Öltanker hat große Mengen Erdöls verloren. Die Küste wurde mit Erdöl verschmutzt. Zur Säuberung werden viele Leute eingesetzt, dennoch dauert die Säuberung lange Zeit und die Verschmutzung ist noch jahrelang vorhanden. Die Federn von Vögeln verkleben und viele andere Tiere sterben an Sauerstoffmangel und Vergiftungen. (Experiment 1) |
| Beim Lottospiel wählt man aus den Zahlen 1 bis 49 sechs Zahlen aus. Bei der Ziehung werden dann Kugeln mit den Zahlen von 1 bis 49 beschriftet und in eine Lostrommel gegeben. Nacheinander werden sechs Zahlen und eine Zusatzzahl gezogen. Um "sechs Richtige" zu erhalten, braucht man eine Menge Glück. Am Lottospiel verdient vor allem der Staat über Steuern und Abgaben auf das Lottospiel. (Experiment 1) |
| ln manchen Fernsehsendungen wird dazu aufgefordert, möglichst oft für einen bestimmten Kandidaten anzurufen oder einfache Fragen zu beantworten. Dabei wird man in den meisten Fällen nicht durchgestellt. Es ist nur eine Ansage vom Band zu hören: "Hallo! Vielen Dank, dass Sie uns angerufen haben. Leider haben Sie dieses Mal kein Glück gehabt. Versuchen Sie es gern noch mal. Dieser Anruf kostet Sie 50 Cent." (Experiment 1) |
| Jeden Montagmorgen, Dienstagmorgen und Donnerstagmorgen fuhren sie miteinander zu Schule. Wenn es ihm gelang, bis zu ihrer Haltestellen den Platz neben sich freizuhalten, saßen sie nebeneinander. In dem überfüllten Bus besaßen sie gemeinsam eine einsame Insel: Ein Sitz-Insel oder eine Steh-Insel, wo sie redeten und redeten und redeten. (Experiment 2) |
| Die Ausbildung im Beruf Maskenbildner/in ist noch nicht bundesweit einheitlich geregelt: Von Betrieb zu Betrieb und von Schule zu Schule kann es Unterschiede geben. Zurzeit kann man den Beruf bei Theatern, Fernsehanstalten und privaten Schulen machen. Die Ausbildung dauert zwei bis vier Jahre. Oft wird eine abgeschlossene Berufsausbildung zum/zur Friseur/in vorausgesetzt. (Experiment 2) |
| Achim schob sich halb aus dem Bett. Fünf nach eins. Wieder mal zu spät. Er starrte gegen die Zimmerdecke. Weiß. Nichts. Ein unbeschriebenes Blatt Papier, ein ungemaltes Bild, eine tonlose Melodie, ein ungesagtes Wort, ungelebtes Leben. Eine halbe Körperdrehung nach rechts, ein Fingerdruck auf den Einschaltknopf seiner Anlage. Manchmal brachte Musik ihn hoch. (Experiment 2) |
| Die mittlerweile an Wahnsinn grenzende Tierproduktion fordert dabei nicht nur von den Masttieren, sondern auch von der Umwelt einen hohen Preis. Boden und Gewässer werden durch die enormen Güllemengen vergiftet, die bei der hohen Tierkonzentration in einem Zuchtbetrieb anfallen. Stickstoffverbindungen gelangen über Bäche und Flüsse in die Meere und Seen und sind für das vermehrte Algenwachstum verantwortlich. (Experiment 2) |
| Dialekte sind lokale oder regionale Sonderformen einer Sprache. Der auf das lateinische "dialectos" (=Ausdrucksweise) zurückgehende Begriff wurde im 17. Jahrhundert durch Philipp von Zesen mit Mundarten eingedeutscht. Dialekte gehören zu den Sprachvarietäten. Sprachwissenschaftler verwenden den Begriff, um mögliche Vorurteile gegenüber Dialekten zu vermeiden. Prinzipiell wird bei Dialekten einer Sprache eine gegenseitige Verständlichkeit bewahrt. Doch können die Abweichungen in Aussprache, Wortschatz und Teilen der Grammatik sowie in Bedeutung und Gebrauch der Wörter sehr groß sein. (Experiment 2) |
